# Editorial: Structural Transitions in the Health Care Systems in Times of Uncertainty

**DOI:** 10.3389/fpubh.2018.00233

**Published:** 2018-08-29

**Authors:** Piotr Romaniuk, Tomasz Holecki

**Affiliations:** ^1^Department of Health Policy, School of Public Health in Bytom, Medical University of Silesia in Katowice, Bytom, Poland; ^2^Department of Health Economics and Health Management, School of Public Health in Bytom, Medical University of Silesia in Katowice, Bytom, Poland

**Keywords:** health systems, transition, health reforms, health care management, health care assessment

The health care systems nowadays are complex structures, strongly engaged in bidirectoral influences with other spheres of social and economic life. Health systems, at the same time, has reached a status of focal area of public responsibility, being designed to answer essential population's needs and consuming a growing part of public expenditure. For that reason a suitable design of the systems maximizing their effectiveness remain of crucial importance for the general socio-economic systems in all contemporary countries. The issue, however, stays out of impact of simple one-dimensional solutions, being stressed by constant and diverese in nature pressure arising of internal and external factors. Constantly evolving internal structure becomes more and more complicated and susceptible to turmoils caused by competing interest groups. On the other hand, the external environment adds more concerns. Economic growth or decline may strongly affect systemic ability to meet population's health needs and ability to stabilize internal structure. Additionally, the social needs and expectations to be addressed remain dynamic in nature, showing tendencies to grow, but also transform, following the changing demographic structure. Current and future challenges related to health systems optimization undoubtedly include demographic transformation, in particular those related to the aging of the population, and the ability to cover at the expected level the demand for goods and services of the health care market. In the face of these rapid and inconsistent changes the internal and external health system environment is experiencing, there is a need to gain ability to seize opportunities and minimize emerging threats.

Thus, the presented research topic explores the issues of structural transitions in the health care systems in times of uncertainty, being inscribed into the broad context of tasks related to protecting and improving the health of the populations at the macro- and microeconomic level. It aims to explore pressure on health care systems, which arises of the described processes, but first of all—it makes and effort to explore the response on the systemic side, in terms of structural, organizational, and financial dimensions. This refers to both the general solutions applied on the level of health system, and to the level of health care organizations, public bodies, or even individuals.

Papers published in the topic are rising issues of health system evaluation (Romaniuk et al.), implementation of particular systemic solutions, which are aimed to answer particular health needs (Holecki et al.), i.e., oncological care, measures of systemic sustainability and equality (Dlouhý), as well as the potential solutions to minimize them using different instruments. The epidemiological and economy-related issues has been also raised, including morbidity, disability, and work absenteeism (Jakovljevic et al.), along with economic and social problems of out-of-pocket health spendings (Grima et al.). The broadness and complexity of health systems issues is reflected by the problem of public communication (Syrkiewicz-Świtała et al.) and the discussion on deliverance of particular instruments to provide health promotion services by the regional administration handling responsibility for public health.

Authors engaged in the topic addressed diverse aspects of health system performance and assessment. This remains in line with the interdisciplinary concept of health sciences, which can be perceived through the multitude of problems existing in public health, but also in context of multiplicity of methods to solve them. We truly believe the proposed scope of problems raised in the topic will be a valuable contribution to the international discourse and will gain a deserved interest, but also critical response, from an extensive audience worldwide.

## Author contributions

PR outlined main theses and prepared final version of the paper. TH drafted the paper.

### Conflict of interest statement

The authors declare that the research was conducted in the absence of any commercial or financial relationships that could be construed as a potential conflict of interest.

